# Class IIa histone deacetylase (HDAC) inhibitor TMP269 suppresses lumpy skin disease virus replication by regulating host lysophosphatidic acid metabolism

**DOI:** 10.1128/jvi.01827-24

**Published:** 2025-01-22

**Authors:** Pengyuan Cheng, Xiangwei Wang, Shasha Wang, Shanhui Ren, Zhengji Liang, Ke Guo, Min Qu, Xuelian Meng, Yongxi Dou, Xiangping Yin, Yuefeng Sun

**Affiliations:** 1 State Key Laboratory for Animal Disease Control and Prevention, College of Veterinary Medicine, Lanzhou Veterinary Research Institute, Chinese Academy of Agricultural Sciences, Lanzhou University 12426 , Lanzhou, China; University of Kentucky College of Medicine, Lexington, Kentucky, USA

**Keywords:** LSDV, TMP269, lysophosphatidic acid, MEK/ERK signaling pathway, viral replication, innate immune response

## Abstract

Lumpy skin disease virus (LSDV) infection poses a significant threat to global cattle farming. Currently, effective therapeutic agents are lacking. TMP269, a small molecule inhibitor of class IIa histone deacetylase inhibitor, plays a vital role in cancer therapy. In this study, we demonstrated that TMP269 treatment inhibits the early-stage replication of LSDV in a dose-dependent manner. RNA sequencing data revealed that metabolism-related signaling pathways were significantly enriched after LSDV infection. Furthermore, untargeted metabolomics analysis revealed that lysophosphatidic acid (LPA), a key metabolite of the glycerophospholipid pathway, was upregulated following LSDV infection and downregulated after TMP269 treatment. In addition, exogenous LPA promotes LSDV replication by activating the mitogen-activated protein kinase (MEK)/extracellular-signal-regulated kinase (ERK) signaling pathway and suppressing the host’s innate immune response. Furthermore, treatment with the LPA receptor inhibitor Ki16425 suppressed LSDV replication and promoted the host’s innate immune response. These findings suggest that LSDV infection can induce LPA expression and aid viral activation of the MEK/ERK signaling pathway and escape of the host’s innate immune response, whereas TMP269 treatment can inhibit LPA production and limit its promotion of LSDV replication. These data identified the antiviral mechanism of TMP269 and a novel mechanism by which LSDV inhibits host innate immune responses, providing insights into the development of new preventive or therapeutic strategies targeting altered metabolic pathways.

**IMPORTANCE:**

Lumpy skin disease virus (LSDV) poses a significant threat to global cattle farming. Owing to insufficient research on LSDV infection, pathogenesis, and immune escape mechanisms, prevention and control methods against LSDV infection are lacking. Here, we found that TMP269, a class IIa histone deacetylase inhibitor, significantly inhibited LSDV replication. We further demonstrated that TMP269 altered LSDV infection-induced host glycerophospholipid metabolism. In addition, TMP269 decreased the accumulation of lysophosphatidic acid (LPA), a key metabolite in glycerophospholipid metabolism, induced by LSDV infection, and exogenous LPA-promoted LSDV replication by activating the mitogen-activated protein kinase (MEK)/extracellular-signal-regulated kinase (ERK) signaling pathway and suppressing the host innate immune response. Our findings identified the antiviral mechanism of TMP269 and a novel mechanism by which LSDV manipulates host signaling pathways to promote its replication, offering insights into the development of novel antiviral agents against LSDV infection.

## INTRODUCTION

Lumpy skin disease (LSD), caused by the LSD virus (LSDV), is an acute and subacute infectious disease. Cattle infected with LSDV can experience permanent skin damage, reduced milk production, abortion, infertility, and mastitis (1, 2), posing a threat to cattle farming and causing significant economic losses worldwide (3). After its report in Zambia and South Africa in 1929, LSD has gradually spread northward to Israel, from Africa to the Middle East, and rapidly and widely throughout Russia and Asia since 2013. In China, the first occurrence of LSDV infection was reported in Qapqal Xibe Autonomous County, Ili Kazakh Autonomous Prefecture of Xinjiang located in northwestern China in August 2019, spreading to the southeastern coastal areas of China within the following 2 years (4). However, no specific antiviral drugs are currently available.

LSDV is a double-stranded DNA virus, belonging to the *Capripoxvirus* genus of the *Poxviridae* family. It has a genome size of 151 kb and encodes 156 predicted proteins involved in viral mRNA, nucleotide metabolism, DNA replication, protein processing, virion structure and assembly, viral virulence, and infection (5). Owing to insufficient research on LSDV infection, pathogenesis, and immune escape mechanisms, prevention and control methods against LSDV infection are lacking. Therefore, to prevent LSDV infection epidemics, exploring new LSDV prevention and treatment strategies is urgently needed.

Histone deacetylases (HDACs) are a family of enzymes that catalyze the deacetylation of acetylated proteins and play a key role in regulating the acetylation and signal transduction. To date, 18 HDACs have been identified and classified into four (I–IV) based on their structures. Several studies have shown that viral replication involves HDAC deacetylation, by which the host’s innate immune response is regulated. HDAC inhibitors (HDACis) were initially considered effective anticancer agents; however, recent findings showed that HDACis are also involved in regulating viral replication. For example, suberoylanilide hydroxamic aci, a class I, II, and IV HDACis, has been approved by the United States Food and Drug Administration for the treatment of malignant metastatic cutaneous T-cell lymphoma (6), with positive or negative regulatory effects on viral replication (7–10). TMP269 is a selective Class IIa HDACi that targets HDAC4/5/7/9 (11), which plays a vital role in the treatment of cancer and neurological diseases (12, 13). In our previous study, we found that TMP269 significantly inhibited rabies virus replication (14); however, its effect on other viruses has not been described.

In this study, we investigated the effect of TMP269 on LSDV replication. We found that TMP269 can inhibit LSDV replication during the early stage of LSDV life cycle in a dose-dependent manner. To explore the mechanism of TMP269 inhibiting LSDV replication, RNA-seq and untargeted metabolomics analyses were performed, demonstrating that TMP269 altered host glycerophospholipid metabolism induced by LSDV infection. In addition, TMP269 decreased the accumulation of lysophosphatidic acid (LPA), a key metabolite in the glycerophospholipid metabolism, induced by LSDV infection, and exogenous LPA addition was found to promote LSDV replication through activating the mitogen-activated protein kinase (MEK)/extracellular-signal-regulated kinase (ERK) signaling pathway and suppressing the host innate immune response. These data illustrate the antiviral mechanism of TMP269 and a novel mechanism by LSDV to manipulate host signaling pathways to promote their own replication, which will provide insights for the development of new preventive or therapeutic strategies targeting the altered metabolic pathways.

## MATERIALS AND METHODS

### Cells and virus

Mardin–Darby bovine kidney (MDBK), African green monkey kidney (Vero), and baby hamster kidney (BHK)-21 cells were cultured in Dulbecco’s modified Eagle’s medium (DMEM; JSBio, 66001) supplemented with 10% fetal bovine serum (FBS) (Cell-Box, AUS-01S-02) with 1% penicillin-streptomycin (Gibco/Thermo Scientific, 10378016) in a humidified incubator at 37°C with 5% CO_2_. The LSDV/FJ/CHA/2021 (GenBank: OP752701.1) and GTPV/AV41 strains were isolated and stored in the biosafety level-3 (BSL-3) laboratory of the Lanzhou Veterinary Research Institute (LVRI) of the Chinese Academy of Agricultural Sciences (CAAS). All LSDV-related experiments were conducted in the BSL-3 laboratory of the LVRI following standard protocols and biosafety regulations provided by the Institutional Biosafety Committee.

### Antibodies and reagents

The antibodies used in this study were as follows: β-tubulin mouse monoclonal antibody (mAb) (HC101-01, 1:3,000; TransGen Biotech), ERK1/2 Ab (AF0155, 1:1,000; Affinity), Phospho-ERK1/2 Thr202/Tyr204 Ab (AF1015, 1:1,000; Affinity), and ORF029 mouse mAb and ORF123 mouse mAb, which were prepared and provided by our laboratory. The secondary antibodies used were goat anti-rabbit IgG H&L (HRP) (31460, 1:5,000; Thermo Fisher Scientific), goat anti-mouse IgG H&L (HRP) (31430, 1:5,000; Thermo Fisher Scientific), and CoraLite488-conjugated goat anti-mouse IgG (H+L) (SA00013-1, 1:1,000; Proteintech). Polyvinylidene fluoride (PVDF) membranes were purchased from Merck Millipore (Darmstadt, Germany). 4′,6-Diamidine-2′-phenylindole (DAPI) dihydrochloride was purchased from Beyotime Biotechnology (C1002, 1:5,000; Beyotime, Shanghai, China) . TMP269 was purchased from Selleck Chemicals (Houston, TX, USA). The LPA was purchased from Sigma-Aldrich (St. Louis, MA, USA). Ki16425 cells were purchased from Apex Bio (Houston, TX, USA).

### Cytotoxicity assay

Cell viability was assessed using the CCK-8 (Solarbio, China, CA12010) according to the manufacturer’s instructions. Briefly, MDBK cells were seeded in 96-well plates and incubated for 24 h before the addition of different concentrations of reagents to each well. After 24 h of incubation, 10 µL CCK-8 reagent was added to each well and incubated for an additional 2 h at 37°C. Finally, the absorbance was measured at 450 nm using a microplate reader.

### DNA/RNA extraction and qRT-PCR

Total DNA was isolated from cultured cells using a DNA extraction kit (TIANGEN, Beijing, China). Total RNA was isolated from cultured cells using Total RNA Extraction Reagent (Vazyme, Nanjing, China) and reverse transcribed to cDNA using a StarScript III RT Kit (GenStar, Beijing, China). Quantitative real-time PCR (qRT-PCR) experiments were performed using SGExcel Fast SYBR Mixture (Sangon Biotech, Shanghai, China). The data were presented as the fold change normalized to GAPDH based on the 2−ΔΔCT method (15). Table 1.

**TABLE 1:** Primer sequences used in this study

Primer	Sequence (5′→3′)
ORF061-F (RT-qPCR)	CAACCGGAACAACGACAAAC
ORF061-R (RT-qPCR)	ACTCGCCTTTGTGGTTACAT
Bovine-IFN-β-F (RT-qPCR)	CCTGTGCCTGATTTCATCATGA
Bovine-IFN-β-R (RT-qPCR)	AAAGAGCTGTGGTGGAGAAACAC
Bovine-ISG54-F (RT-qPCR)	CAAGAACCCAGAGTTCGCCT
Bovine-ISG54-R (RT-qPCR)	GCCAATGGGGCTTTTTCCAG
Bovine-ISG56-F (RT-qPCR)	GAATTATGAACGGGCCAAAG
Bovine-ISG56-R (RT-qPCR)	CCCTCCAGGCGATAGACA
Bovine-GAPDH-F (RT-qPCR)	CGATGCCCCCATGTTTGTGA
Bovine-GAPDH-R (RT-qPCR)	GATATTCTGGGCAGCCCCTC
Bovine-IFIT3-F (RT-qPCR)	ATCACCTCCTAAAGAACAGCCCTTC
Bovine-IFIT3-R (RT-qPCR)	TGCTCCATTTCCTCACTGCCTTC
Bovine-IL21R-F (RT-qPCR)	CTGCTCCAACCTCGTCTGCTAC
Bovine-IL21R-R (RT-qPCR)	GGTGACTTCATCCTCCAGTTCTCC
Bovine-MX2-F (RT-qPCR)	TCCGCTGGTGCTGAAACTGAC
Bovine-MX2-R (RT-qPCR)	ATGATGTTCTGGGCTCTCCGAATC
Bovine-SMAD9-F (RT-qPCR)	GAGGTCTATGCCGAGTGTGTGAG
Bovine-SMAD9-R (RT-qPCR)	GGGTGGAAGCCGTGCTGATAG
Bovine-MMP9-F (RT-qPCR)	TGGCTTGCTGCTCTGCTGTC
Bovine-MMP9-R (RT-qPCR)	TGGTGAGGTTGGTTCGTGGTTC
Bovine-CXCL3-F (RT-qPCR)	CGATGCTGCTCCTGCTCCTG
Bovine-CXCL3-R (RT-qPCR)	GAGGTGAATCCCCTGCAAAGTTTG
Bovine-CXCL5-F (RT-qPCR)	AGAGCTGCGTTGTGTGTGTTTAAC
Bovine-CXCL5-R (RT-qPCR)	GTCCAGACAGACTTCCCTTCCATTC
Bovine-C-MYC-F (RT-qPCR)	CAGCCACAGCAAACCTCCTCAC
Bovine-C-MYC-R (RT-qPCR)	CCTGCCACTGTCCAACTTAGCC
Bovine-ELK-1-F (RT-qPCR)	AGCATTCACTTCTGGAGCACACTG
Bovine-ELK-1-R (RT-qPCR)	GCTGCCACTGGACGGAAACTG
Bovine-UBF-F (RT-qPCR)	CTGAACCACCTGCCGCTGAAG
Bovine-UBF-R (RT-qPCR)	CCTGCTTCTTGTAGTGCTCCTTCTG

### Indirect immunofluorescence assay

MDBK cells were fixed with 4% paraformaldehyde for 20 min, permeabilized with 0.2% Triton X-100 for 5 min, and blocked with 5% bovine serum albumin for 30 min. The corresponding antibody labeled as the primary antibody was incubated at 4°C for 8–12 h. After washing with phosphate-buffered saline (PBS) thrice for 5 min each, the cells were incubated with secondary antibodies for 1 h each at room temperature and were stained with DAPI for 10 min. Finally, the cells were observed under a fluorescence microscope.

### Western blotting

Protein lysates were obtained by blocking cells with radio immunoprecipitating assay lysis buffer. Protein lysates were separated by sodium dodecyl sulfate-polyacrylamide gel electrophoresis and transferred onto PVDF membranes. After this, the PVDF membranes were blocked with 5% skimmed milk for 1 h at room temperature and then incubated with primary antibodies overnight at 4°C. Then, the PDVF membranes were washed thrice for 10 min each and were incubated with horseradish peroxidase-conjugated secondary antibody for 1 h at room temperature. Finally, we visualized and analyzed the results using a gel imager (GE-AI600, USA).

### TCID_50_ assay

A 50% tissue culture infectious dose (TCID_50_) assay was used to determine the viral titers. MDBK cells were suspended in a 96-well plate, and once the cell density reached over 90% confluence, the cells were infected. The supernatants collected from each group of cells were serially diluted in serum-free DMEM, from 10^−1^ to 10^−8^ in 10-fold increments. Furthermore, 100 µL of each diluted sample was added to each well of the 96-well plate and was incubated at 37°C for 1 h. After removing the inoculum, the cells were continuously cultured in DMEM supplemented with 1% FBS for 72–120 h. The cytopathic effect was recorded daily, and the TCID_50_ was calculated using the Reed-Muench method (16).

### Enzyme-linked immunosorbent assay (ELISA)

The collected cell culture supernatant samples were centrifuged, and the ELISA kits were equilibrated to room temperature. Standards were diluted according to the manufacturer’s instructions and added to a 96-well plate along with cell culture supernatant samples. The following steps were performed according to the manufacturer’s protocol: incubation with the horseradish peroxidase conjugate, washing, color development, and termination. The absorbance was measured at 450 nm using an ELISA microplate reader (Thermo Fisher Scientific, Waltham, MA, USA) to calculate the concentration of the target analytes. In this experiment, bovine-specific ELISA kits (for LPA, lysophosphatidylcholine [LPC], and IFN-β) were purchased from mlbio (Shanghai, China).

### Time-of-addition assay

TMP269 was added at a concentration of 20 µM at 6 h or 3 h before LSDV [0.5 multiplicity of infection (MOI)] infection and at 0, 2, 4, and 6 h after infection and was maintained in the medium. After 24 h post-infection, cell samples or cell culture supernatants were collected for indirect immunofluorescence, Western blotting (WB), TCID_50_, and qRT-PCR.

### Effect of TMP269 on LSDV life cycle

The experimental procedures were as follows: (i) adsorption: MDBK cells were pretreated with the indicated concentrations of TMP269 at 37°C for 1 h. Subsequently, a 15 min pre-cooling step on ice was performed. Thereafter, the cells were inoculated with LSDV (1 MOI) at 4°C for 1 h, washed with PBS to remove unbound viruses, and were lysed for qRT-PCR. (ii) Internalization: MDBK cells were initially infected with LSDV (1 MOI) at 4°C for 2 h, then washed with PBS to remove unbound viruses. Thereafter, different concentrations of TMP269 were subsequently added, and the cells were further incubated at 37°C for 1 h and lysed for qRT-PCR. (iii) Replication: MDBK cells were initially infected with LSDV (1 MOI) at 4°C for 2 h, then washed with PBS to remove unbound viruses. Thereafter, different concentrations of TMP269 were subsequently added, and the cells were further incubated at 37°C for 12 h and lysed for qRT-PCR.

### Transcriptome RNA-seq

MDBK cells were cultured in 6-well plates and infected with LSDV (0.5 MOI) only or LSDV (0.5 MOI) treated with 20 µM of TMP269 for 24 h. The total RNA of cell samples was extracted using TRIzol Reagent. RNA sequencing and analysis were conducted by Azenta Life Sciences (Suzhou, China). Libraries with different indices were multiplexed and loaded on an Illumina HiSeq/Illumina Novaseq/MGI2000 instrument for sequencing using a 2 × 150 paired-end configuration according to the manufacturer’s instructions. After quality control, reference genome sequences and gene model annotation files of related species were downloaded from the UCSC, National Center of Biotechnology Information, and ENSEMBL genome websites. Hisat2 (v2.2.1) was used to index the reference genome sequences. High-quality clean data were aligned to the reference genome using Hisat2 (v2.2.1) software. Differential expression analysis was performed to identify differentially expressed genes (DEGs) in the different groups. Functional annotation and pathway analysis of the DEGs were performed using the Gene Ontology and Kyoto Encyclopedia of Genes and Genomes (KEGG) databases.

### Untargeted metabolomics analysis using the LC-MS method

MDBK cells were cultured in 6-well plates and infected with LSDV (0.5 MOI) (dimethyl sulfoxide [DMSO] group), LSDV (0.5 MOI) treated with 20 µM of TMP269 (TMP269 group), or DMSO only (MOCK group) for 24 h and were washed with pre-chilled PBS. Cells (1 × 10^7^) from each group were collected using a cell scraper and were frozen in liquid nitrogen for 5–10 min. The samples were thawed on ice. A 500 µL solution (methanol:water = 4:1, vol/vol) containing internal standard was added into the cell sample and was vortexed for 3 min. Samples were placed in liquid nitrogen for 5 min, dried on ice for 5 min, thawed on ice, and vortexed for 2 min. This freeze–thaw cycle was repeated three times. The sample was centrifuged at 12,000 rpm for 10 min (4°C). A 300 µL of supernatant was collected and placed at −20°C for 30 min. The sample was then centrifuged at 12,000 rpm for 3 min (4°C). Aliquots (200 µL) of supernatant were transferred for liquid chromatography-mass spectrometry (LC-MS) analysis.

The collected supernatant was injected into an HSS T3 column (1.8 µm, 2.1 mm × 100 mm; Waters, Milford, MA, USA) under the conditions of 40°C using Nexera UHPLC LC-30A (LC-30A). The sample volume injected into this system was 4 µL, and the flow rate was 0.4 mL/min. The mobile phase of the system comprised two parts (solution A, water containing 0.1% formic acid; solution B, acetonitrile containing 0.1% formic acid). The gradient conditions of the T3 chromatographic column were as follows: 0 min (95:5 A:B), 2 min (80:20 A:B), 5 min (40:60 A:B), 6 min (1:99 A:B), 7.5 min (1:99 A:B), 7.6 min (95:5 A:B), and 10 min (95:5 A:B).

MS detection utilizes electrospray ionization in both positive and negative ion modes. Data acquisition was performed in information-dependent acquisition mode using Analyst TF 1.7.1 Software (Sciex, Concord, ON, Canada). The source parameters were set as follows: ion source gas 1, 50 psi; ion source gas 2, 50 psi; curtain gas, 25 psi; temperature, 550°C; ion spray voltage floating, 5,000 V or 4,000 V in positive or negative modes, respectively. The time-of-flight MS scan parameters were set as follows: mass range, 50–1,000 Da; accumulation time, 200 ms; and dynamic background subtraction, ON. The product ion scan parameters were set as follows: mass range, 25–1,000 Da; accumulation time, 40 ms; collision energy, 30 or −30 V in positive or negative modes, respectively; collision energy spread, 15; resolution, UNIT; charge state, 1 to 1; intensity, 100 cps; exclude isotopes within 4 Da; mass tolerance, 50 ppm; and maximum number of candidate ions to monitor per cycle, 18.

### Statistical analysis

All results are expressed as the mean ± standard deviation of three independent experiments and analyzed using Student’s *t*-test or one-way analysis of variance with GraphPad Prism 10.1.2 (GraphPad Software Inc., La Jolla, CA, USA). Differences were considered statistically significant at *P* < 0.05. *P < 0.05, **P < 0.01, ***P < 0.001, and ****P < 0.0001.

## RESULTS

### TMP269 significantly inhibits LSDV infection

**Fig 1:**
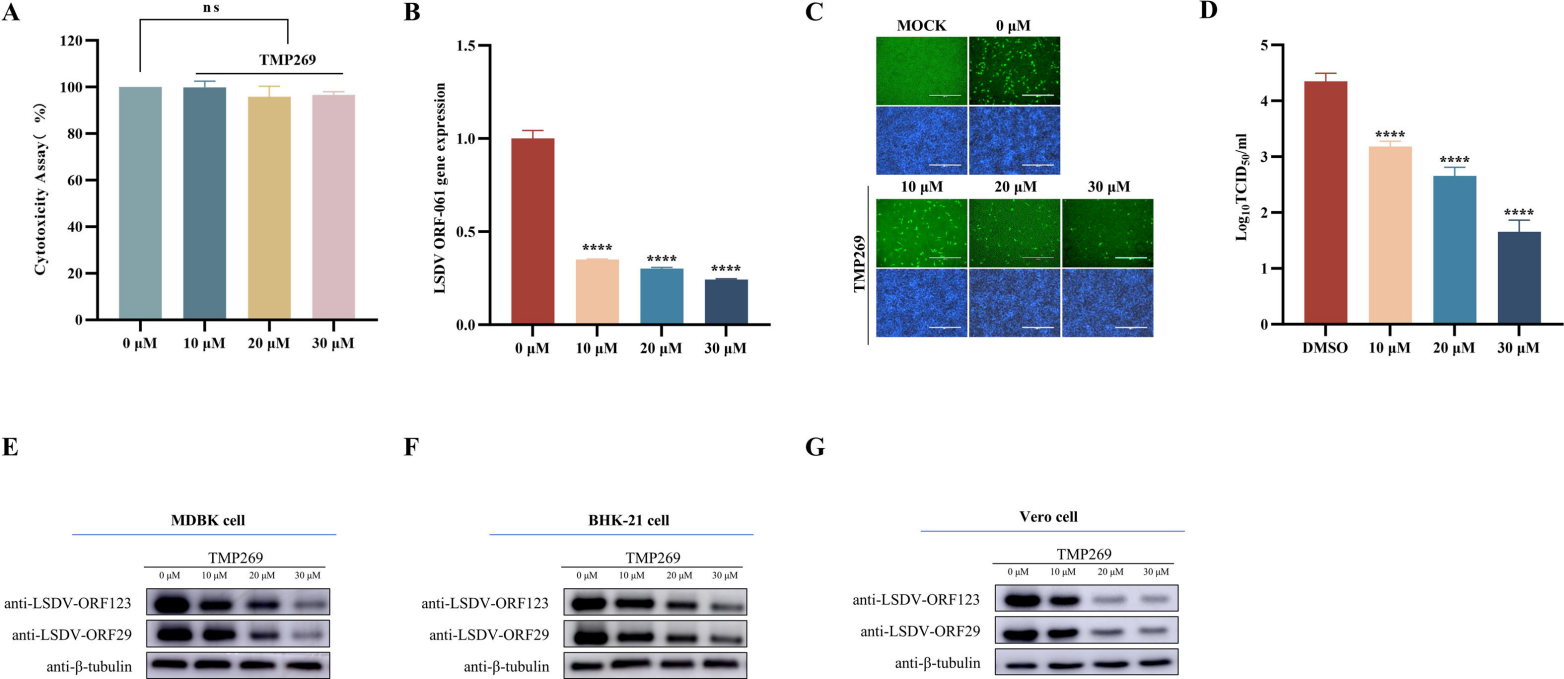
TMP269 significantly inhibits LSDV infection. (**A**) CCK-8 assay was used to detect the toxicity of TMP269 on MDBK cells. (B–E) Effect of TMP269 on LSDV replication. The cells were infected with LSDV (0.5 MOI) and simultaneously treated with different concentrations of TMP269 (10, 20, and 30 µM) for 24 h. The LSDV replication was detected by qRT-PCR (**B**), IFA (**C**), TCID_50_ (**D**), and Western blot (**E**). (**F and G**) Effect of TMP269 on LSDV replication in susceptible cells. BHK-21 cells (**F**) and Vero cells (**G**) were infected with LSDV (0.5 MOI) and simultaneously treated with different concentrations of TMP269 (10, 20, and 30 µM) for 24 h. Western blot was used to detect the replication of LSDV. ***P < 0.001 and ****P < 0.0001.

To investigate the effect of TMP269 on viral replication, cell viability assay was performed using the cell counting kit (CCK)−8. MDBK cells treated with different doses of TMP269 (10, 20, or 30 µM) were analyzed, and the results showed that no obvious cell death was induced at the indicated concentrations (Fig. 1A). Next, we evaluated the effect of TMP269 on LSDV replication. MDBK cells infected with LSDV (0.5 MOI) were treated with indicated concentrations of TMP269. At 24 h post-infection, cell samples were collected for qRT-PCR, WB, immunofluorescence assay (IFA), and TCID_50_ assays to determine viral replication. The results showed that, compared with DMSO treatment, TMP269 treatment significantly inhibits LSDV replication in a dose-dependent manner (Fig. 1B through E). Similar results were obtained for LSDV-infected Vero and BHK-21 cells treated with TMP269 (Fig. 1F and G). In addition, TMP269 significantly inhibited goat pox virus (GTPV) replication (Fig. S1).

### TMP269 exhibits inhibitory effects at different stages of the LSDV life cycle

**Fig 2:**
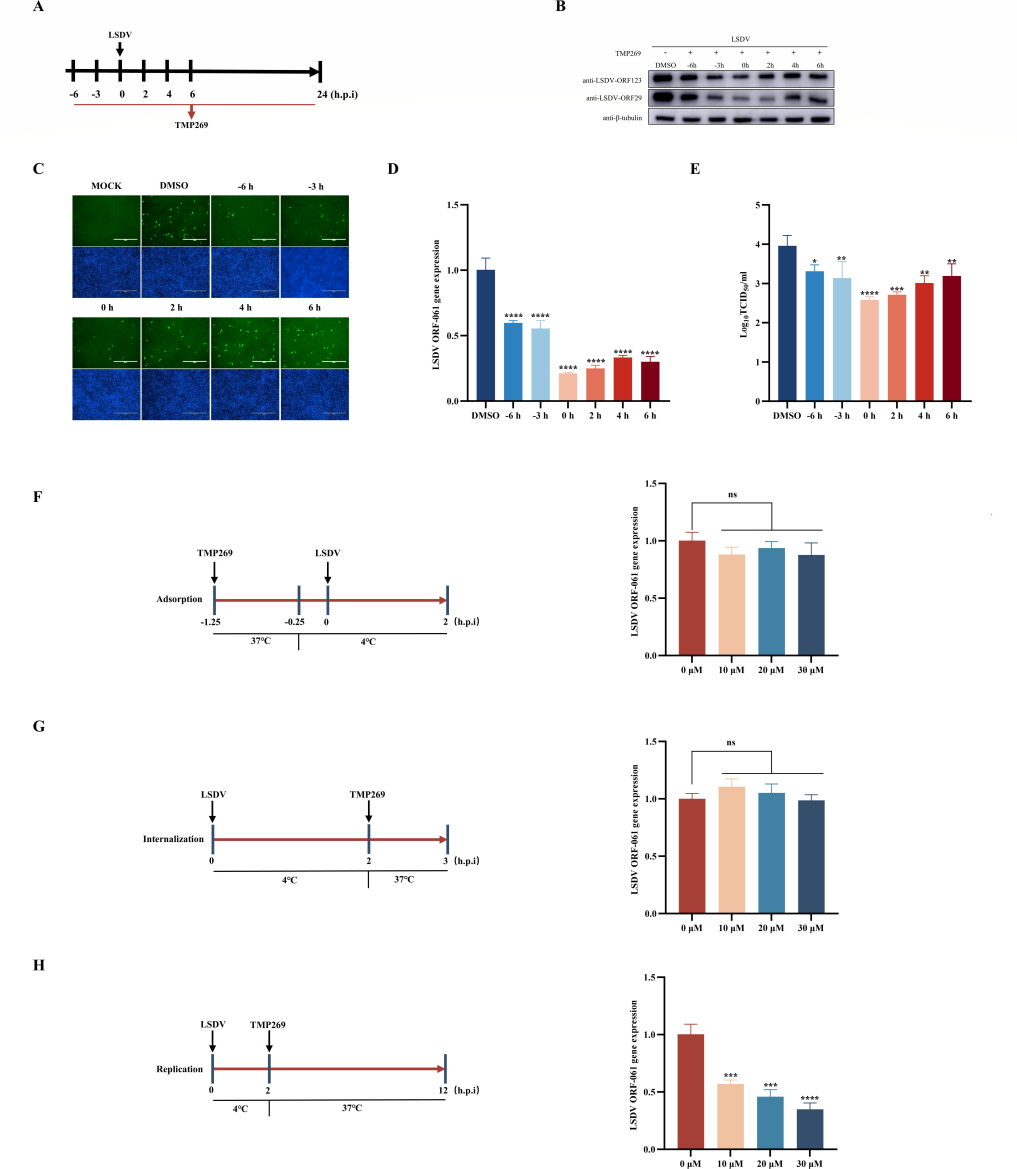
TMP269 exhibits inhibitory effects in the initial stage of LSDV infection. (**A**) Overview of the “time of drug-addition” assay experimental design. TMP269 (20 µM) was added at the indicated time points before or after LSDV infection, and the antiviral effect of TMP269 on LSDV replication was detected via Western blot (**B**), IFA (**C**), qRT-PCR (**D**), and TCID_50_ (**E**). (**F**) Effect of TMP269 on LSDV adsorption stage. MDBK cells were pretreated with the indicated concentrations of TMP269 at 37°C for 1 h. Subsequently, a 15 min pre-cooling step on ice was performed. Thereafter, the cells were inoculated with LSDV (1 MOI) at 4°C for 2 h, washed with PBS to remove unbound viruses, and lysed for qRT-PCR. (**G**) Effect of TMP269 on LSDV internalization stage. Cells were initially infected with LSDV (1 MOI) at 4°C for 2 h and washed with PBS to remove unbound viruses. Thereafter, different concentrations of TMP269 were subsequently added, and the cells were further incubated at 37°C for 1 h and lysed for qRT-PCR. (**H**) Effect of TMP269 on LSDV replication stage. Cells were initially infected with LSDV (1 MOI) at 4°C for 2 h, then washed with PBS to remove unbound viruses. Thereafter, different concentrations of TMP269 were subsequently added, and the cells were further incubated at 37°C for 12 h and lysed for qRT-PCR. ***P < 0.001 and ****P < 0.0001.

To determine which stage of the LSDV life cycle is targeted by TMP269, we conducted a series of experiments. Figure 2A illustrates the methodologies employed using the “time of drug-addition” assay. The MDBK cells were seeded into 12-cell plates and grown to 80% confluence, pretreated with TMP269 (20 µM) for 6 and 3 h, and infected with LSDV (MOI = 0.5) for 24 h without TMP269 treatment, or the cells were infected with LSDV (MOI = 0.5) followed by TMP269 (20 µM) treatment at 0, 2, 4, and 6 h post-infection. At 24 h post-infection, cell samples were collected for WB, IFA, qRT-PCR, and TCID_50_ analysis to determine the viral titer. The results summarized in Fig. 2B through E reveal that TMP269 showed antiviral effects, regardless of whether it was added before or after LSDV infection. When TMP269 was added after LSDV infection, LSDV replication significantly decrease, especially at the early stage, and the inhibitory effect of TMP269 gradually decreased over time. These results demonstrated that TMP269 mainly affects the early stage of LSDV proliferation. Next, we examined the effects of TMP269 on LSDV adsorption, internalization, and replication. As shown in Fig. 2F and G, TMP269 did not affect LSDV adsorption and internalization. TMP269 significantly inhibited LSDV replication (Fig. 2H). Taken together, these results demonstrate that TMP269 inhibits LSDV replication by modulating host-related factors.

### Effect of TMP269 on MDBK cell transcriptomes following LSDV infection

**Fig 3:**
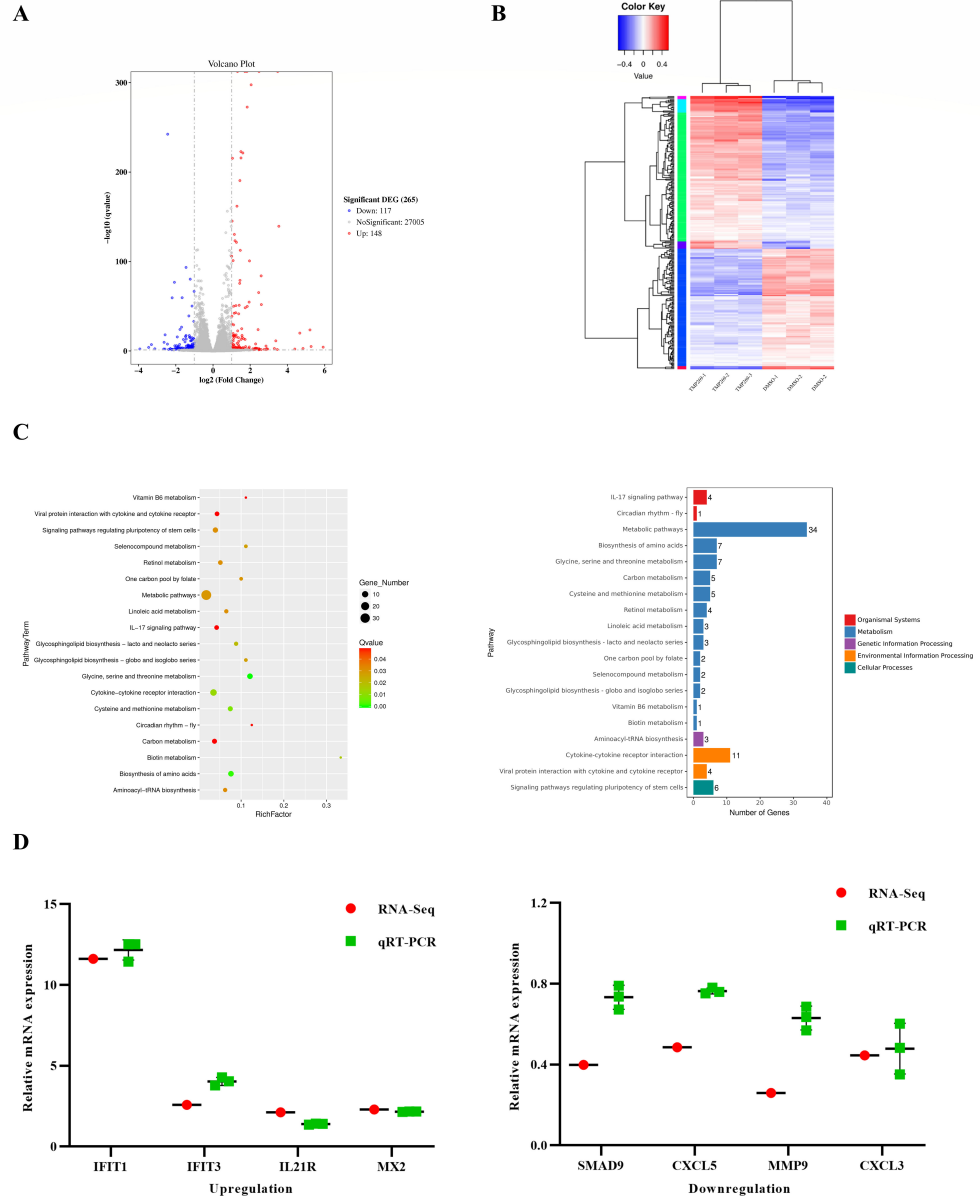
Effect of TMP269 on the transcriptome of MDBK cells following LSDV infection. MDBK cells infected with LSDV (0.5 MOI) were treated with either DMSO (DMSO group) or TMP269 (TMP269 group), and samples were collected 24 h post-infection. (**A**) Volcano plot of differentially expressed genes in the TMP269 group compared with the DMSO group. The red dots represent upregulated genes, and the blue dots represent downregulated genes. (**B**) Clustering diagram of differentially expressed genes in the TMP269 group compared with the DMSO group. Cluster analysis was performed based on log10 (FPKM+1) values, where red indicates a high gene expression, and blue indicates a low gene expression. The color gradient from blue to red represents increasing gene expression levels. (**C**) Differential KEGG Enrichment Scatter Plot and Classification Bar Chart in the TMP269 group compared with the DMSO group. (**D**) The RNA-seq results were verified by qRT-PCR. Red dots represent RNA-seq results, and green squares represent qRT-PCR results.

To identify the molecular mechanisms underlying the antiviral effect of TMP269, the MDBK cells infected with LSDV (MOI = 0.5) for 24 h and treated with DMSO or TMP269 (20 µM) were harvested and subjected to RNA sequencing (three biological replicates per group). The transcripts were filtered based on the following criteria: Q value < 0.05 and |log2 fold change| > 1. Compared with the DMSO treatment group, a total of 265 DEGs (148 upregulated DEGs and 117 downregulated DEGs) were identified (Fig. 3A and B; Table S1) in the TMP269 treatment group after LSDV infection. The top 20 KEGG pathways are shown in Fig. 3C. Among the enriched top 20 pathways, 13 were are related to metabolism, such as metabolic pathways (ko01100), biosynthesis of amino acids (ko01230), and glycine, serine, and threonine metabolism (ko00260), demonstrating that TMP269 may affect the host metabolism process to modulate LSDV proliferation. To verify the RNA-seq results, qRT-PCR was performed on the same RNA samples as those used in the RNA-seq analysis for the eight selected genes. As shown in Fig. 3D, all selected genes exhibited concordant trends, compared with the transcriptome data, supporting the claim that the DEGs identified by the transcriptome are reliable.

### TMP269 treatment changes the metabolite contents and metabolic pathways during LSDV infection

**Fig 4:**
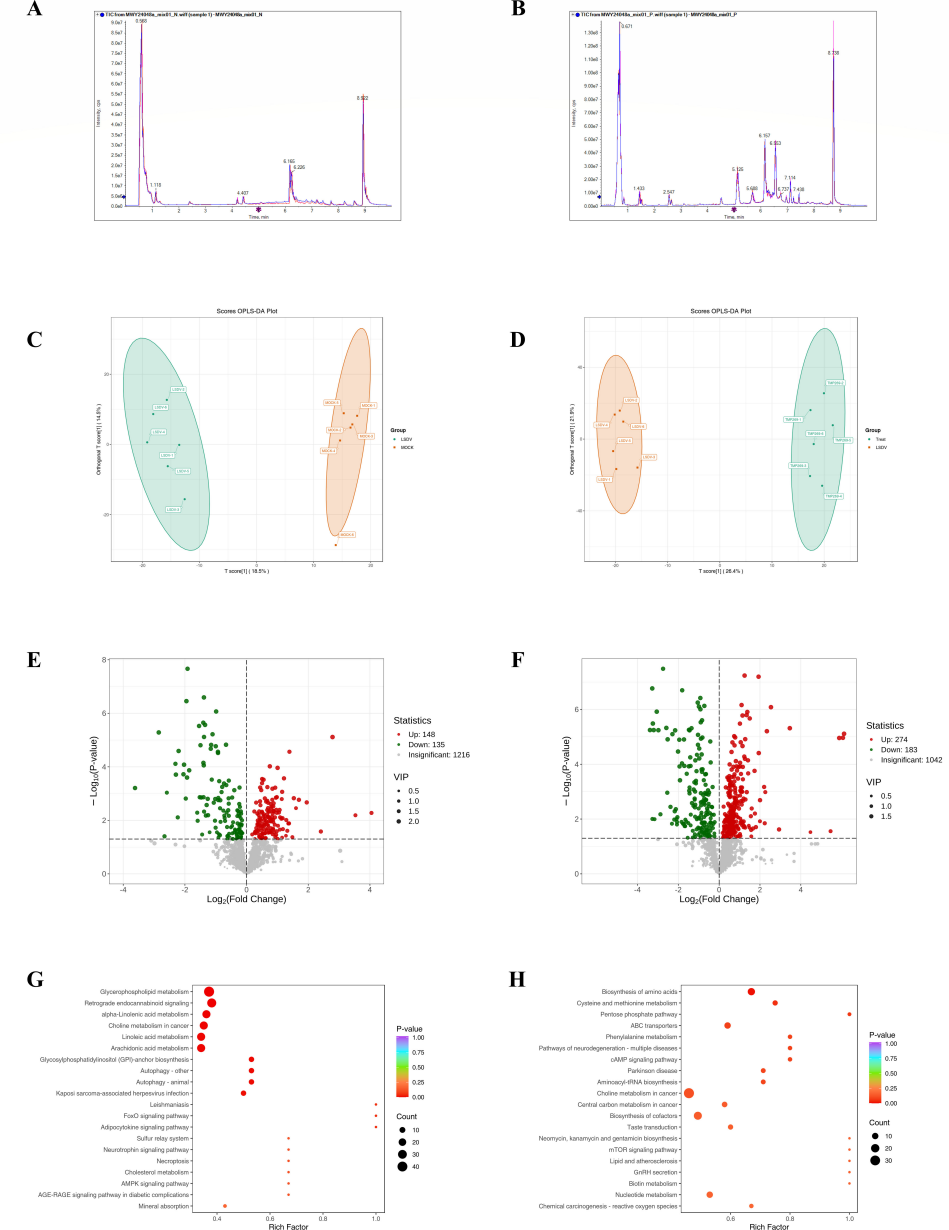
TMP269 treatment changes the metabolite contents and metabolic pathways during LSDV infection. MDBK cells infected with LSDV (0.5 MOI) were treated with either DMSO (DMSO group) or TMP269 (TMP269 group). The MOCK group was treated with DMSO only. (A–D) Quality control (QC) analysis of untargeted metabolomics data. (A) Overlay plot of total ion chromatogram (TIC) in negative ion mode. (B) Overlay plot of TIC in positive ion mode. The results indicate a high overlap in the curves of metabolite detection total ion flow, with consistent retention times and peak intensities. This suggests good signal stability when the mass spectrometer detects the same sample at different times. (C) The OPLS-DA model was generated by comparing the DMSO group with the MOCK group. (D) The OPLS-DA model was generated by comparing the TMP269 group with the DMSO group. (E and F) Volcano plot depicting the differential metabolites in the DMSO group compared with the MOCK group (E), and the TMP269 group compared with the DMSO group (F). Red indicates upregulation, and blue indicates downregulation, whereas gray indicates unchanged. (G and H) KEGG enrichment plot of the top 20 differential metabolites in the DMSO group compared with the MOCK group (G) and the TMP269 group compared with the DMSO group (H). The X-axis represents the rich factor, and the Y-axis represents the pathway. Each bubble represents a metabolic pathway. The larger the bubble, the more enriched the metabolites are. The darker the red, the smaller the *P* value, indicating a greater degree of enrichment significance.

According to the RNA-seq data, many DEGs were enriched in metabolism. To further demonstrate the mechanism underlying TMP269 modulation of LSDV proliferation by regulating host metabolic processes, MDBK cell samples were collected from DMSO-treated and TMP269-treated groups, followed by LSDV infection (0.5 MOI) and the DMSO-treated group (MOCK group) treated only with DMSO for 24 h to perform untargeted metabolomics analysis using liquid chromatography with tandem mass spectrometry (LC-MS/MS). Total ion current (TIC) and orthogonal partial least squares discriminant analysis (OPLS-DA) plots were used to indicate the stability and reliability of the data from the untargeted metabolomic assays (Fig. 4A through D). Different metabolites were obtained through untargeted metabolomic analyses to identify changes in metabolite levels after LSDV infection or treatment with TMP269. The results showed that 283 metabolites were significantly altered in the DMSO group compared with those in the MOCK group, with 148 metabolites significantly increased and 135 significantly decreased (Fig. 4E; Table S2). In addition, 457 metabolites were significantly altered in the TMP269 group compared with the DMSO group, with 274 metabolites significantly increased and 183 significantly decreased (Fig. 4F; Table S3). Subsequently, the KEGG database was used to identify the corresponding metabolic pathways based on the differential metabolites. The results indicate that the differential metabolites in the DMSO group compared with the MOCK group were primarily enriched in the metabolic pathways of glycerophospholipid metabolism, retrograde endocannabinoid signaling, alpha-linolenic acid metabolism, choline metabolism in cancer, linoleic acid metabolism, and arachidonic acid metabolism (Fig. 4G). The differential metabolites in the TMP269 group compared with those in the DMSO group were primarily enriched in choline metabolism in cancer, biosynthesis of cofactors, nucleotide metabolism, ABC transporters, and central carbon metabolism in cancer (Fig. 4H).

### TMP269 treatment decreased the lysophosphatidic-related metabolite expression induced by LSDV infection

**Fig 5:**
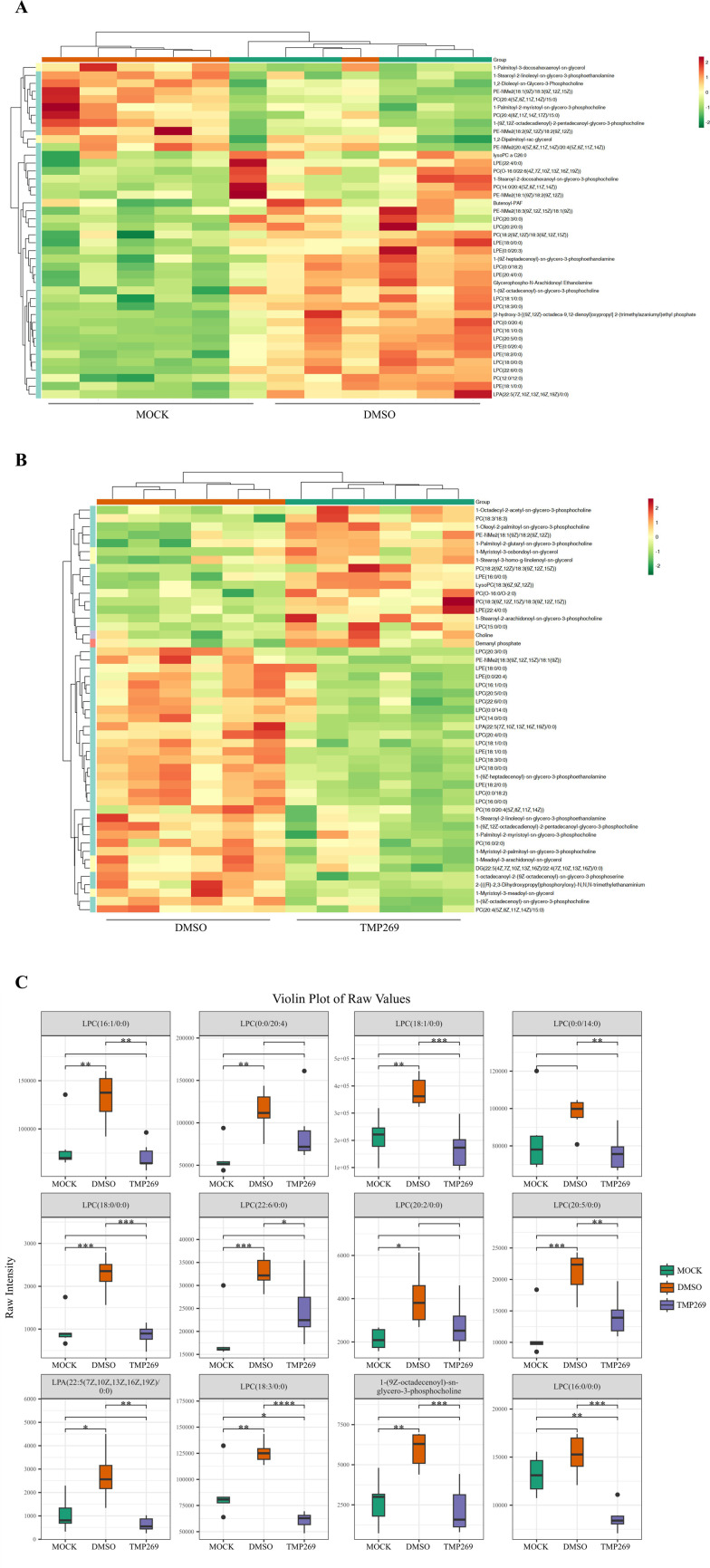
TMP269 treatment decreased the LPA-related metabolites expression induced by LSDV infection. (**A**) Hierarchical clustering heatmap of differential metabolites in the glycerophospholipid pathway between the DMSO group and the MOCK group. (**B**) Hierarchical clustering heatmap of differential metabolites in the glycerophospholipid pathway between the TMP269 and DMSO groups. The heatmap is filled with different colors representing varying relative content standardized values (red indicating high content, green indicating low content) obtained after normalization processing. (**C**) Box plots of significantly altered LPC and LPA in untargeted metabolomics analysis.

**Fig 6:**
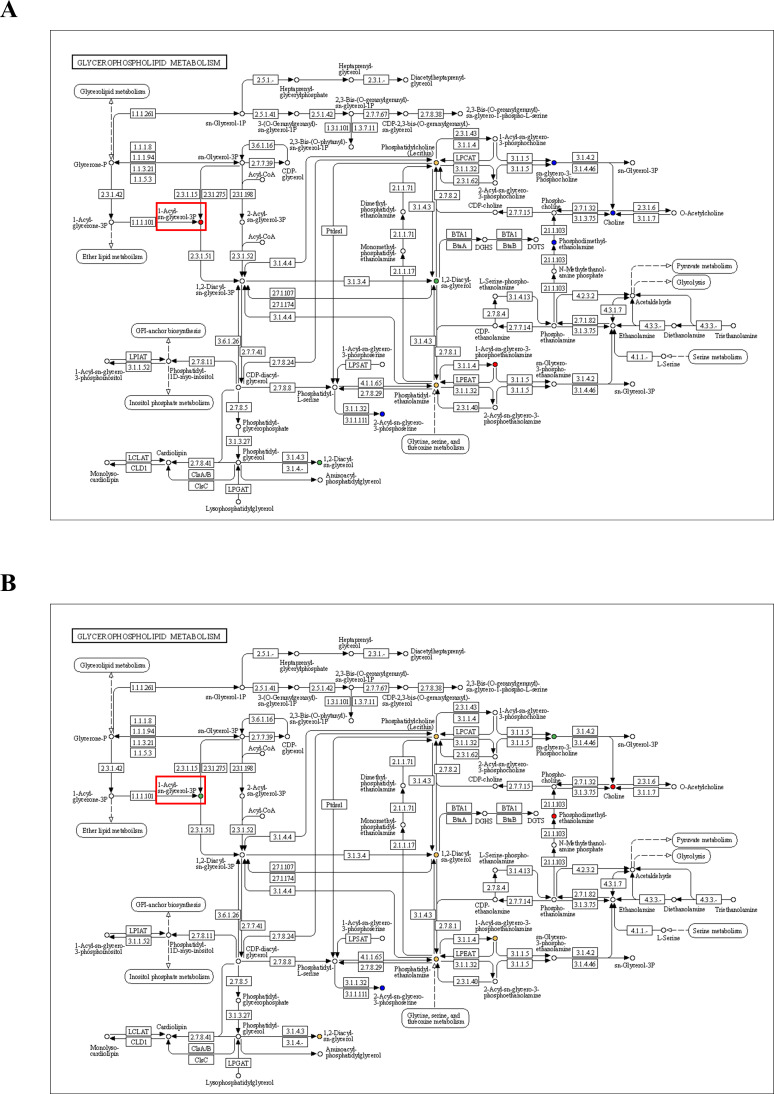
KEGG pathway map of differential metabolites in glycerophosphate pathway. (**A**) KEGG pathway map of differential metabolites in glycerophosphate pathway between the DMSO and MOCK groups. (**B**) KEGG pathway map of differential metabolites in glycerophosphate pathway between the TMP269 and DMSO groups. Red indicates significantly upregulated metabolites in the experimental group, blue represents metabolites detected but not significantly changed, green indicates significantly downregulated metabolites in the experimental group, and orange indicates both upregulated and downregulated metabolites.

To reveal the mechanism by which TMP269 inhibits LSDV replication by regulating the expression of host metabolites, we performed a combined analysis of the untargeted metabolomics results from the comparison between the DMSO and MOCK groups and the TMP269 and DMSO groups. As shown in Fig. S2 and S3, most of the differential metabolites were involved in the glycerophospholipid metabolism pathway in the DMSO group compared with the MOCK group, and in the TMP269 group compared with the DMSO group. Among the differential metabolites involved in the glycerophospholipid metabolism pathway, most metabolites (31/42) were significantly upregulated in the DMSO group compared with those in the MOCK group (Fig. 5A). In contrast, most metabolites (32/49) in the TMP269 group were significantly downregulated compared with those in the DMSO group (Fig. 5B). The LPA and LPC were significantly altered in both groups. Notably, 11 LPCs and one LPA were significantly upregulated in the DMSO group compared with the MOCK group, whereas they were all downregulated in the TMP269 group compared with the DMSO group (Fig. 5C). KEGG pathway annotation of differential metabolites in the glycerophospholipid metabolism pathway showed that LPA (1-Acylsn-glycerol-3P) was significantly increased in the DMSO group compared with the MOCK group (Fig. 6A), while it was significantly decreased in the TMP269 group compared with the DMSO group (Fig. 6B). Taken together, these results demonstrated that TMP269 inhibited LSDV replication by regulating the LPA and LPC metabolism.

### LPA promotes LSDV replication

**Fig 7:**
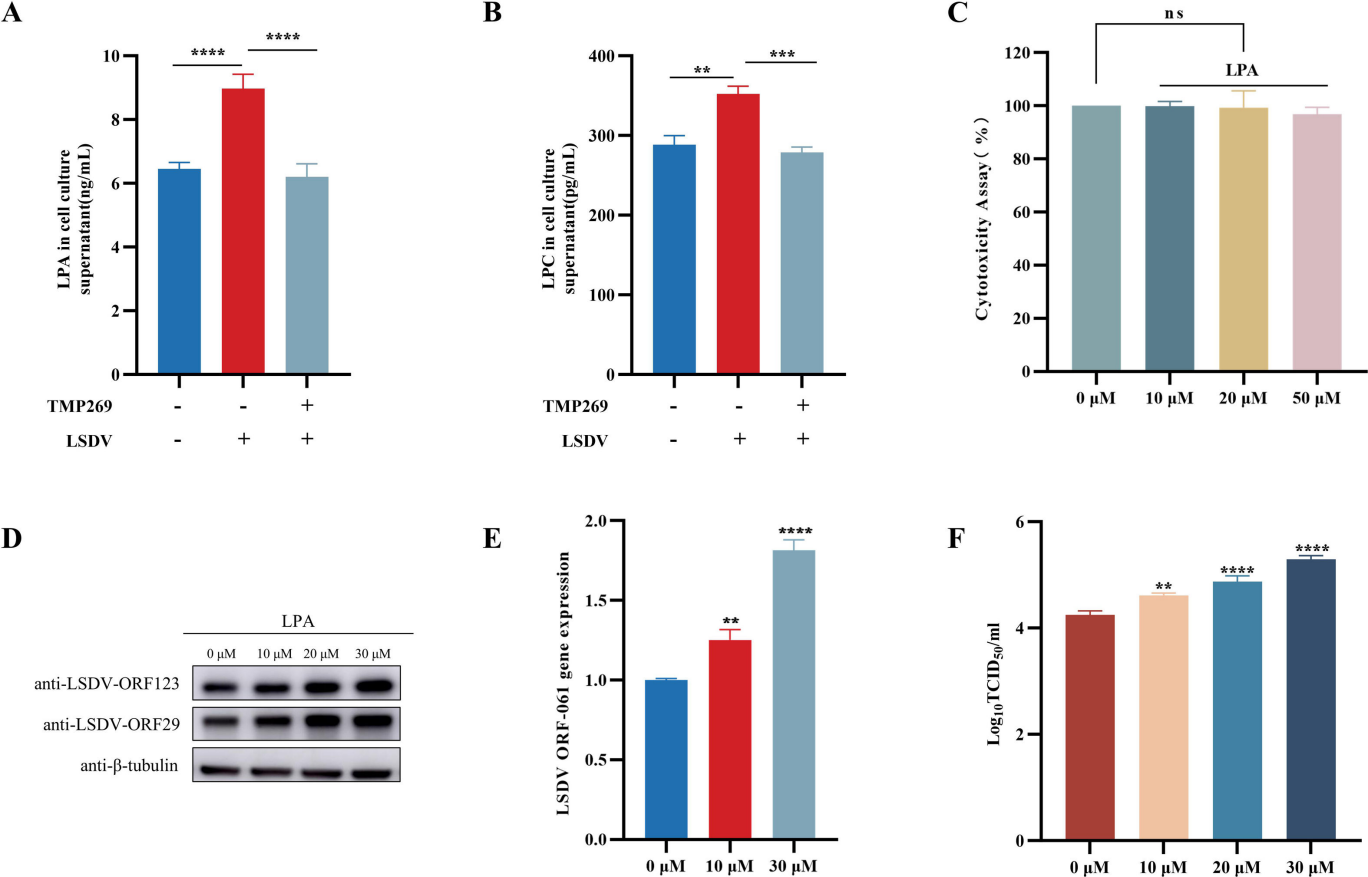
LPA promotes the replication of LSDV. LPA is dissolved in ddH_2_O. (A and B) The expression of LPA and LPC was detected by ELISA. MDBK cells were infected with LSDV (0.5 MOI) and treated with DMSO or 20 µM TMP269 for 24 h, and then the expression of LPA and LPC was detected by ELISA kit according to the manufacturer’s instruction. (C) The cell cytotoxicity of LPA on MDBK cells was determined using a CCK-8 assay. (D–F) Effect of LPA on LSDV replication. MDBK cells were starved in the DMEM medium without FBS for at least 2 h. MDBK cells were pretreated with LPA for 1 h, followed by infection with LSDV (0.5 MOI) for 24 h. The replication of LSDV was detected by Western blot (D), qRT-PCR (E), and TCID_50_ (F). *P < 0.05, **P < 0.01, ***P < 0.001, and ****P < 0.0001.

A previous study revealed that LPC can be hydrolyzed to LPA by autotaxin (17). LPA is a bioactive phospholipid that binds to specific G protein-coupled receptors and exerting various effects (18, 19). In this study, we investigated the role of LPA in LSDV replication. First, we measured the expression of LPA and LPC during LSDV infection and after treatment with TMP269. The results showed that LSDV infection promoted the expression of LPA (Fig. 7A) and LPC (Fig. 7B), which was reduced after treatment with TMP269 (Fig. 7A and B), which was consistent with the results of untargeted metabolomics analysis. We examined the role of LPA in LSDV replication. CCK-8 assay showed that 10, 20, and 50 µM LPA did not affect cell viability (Fig. 7C). Furthermore, the cells were pretreated with different doses of exogenous LPA (10, 20, or 30 µM) for 1 h and infected with LSDV (0.5 MOI). At 24 h post-infection, cell samples were collected for WB, qRT-PCR, and TCID_50_ analysis to determine viral replication. The results showed that LPA treatment significantly promoted LSDV replication in a dose-dependent manner (Fig. 7D through F).

### LPA promotes LSDV replication by activating the MEK/ERK signaling pathway

**Fig 8:**
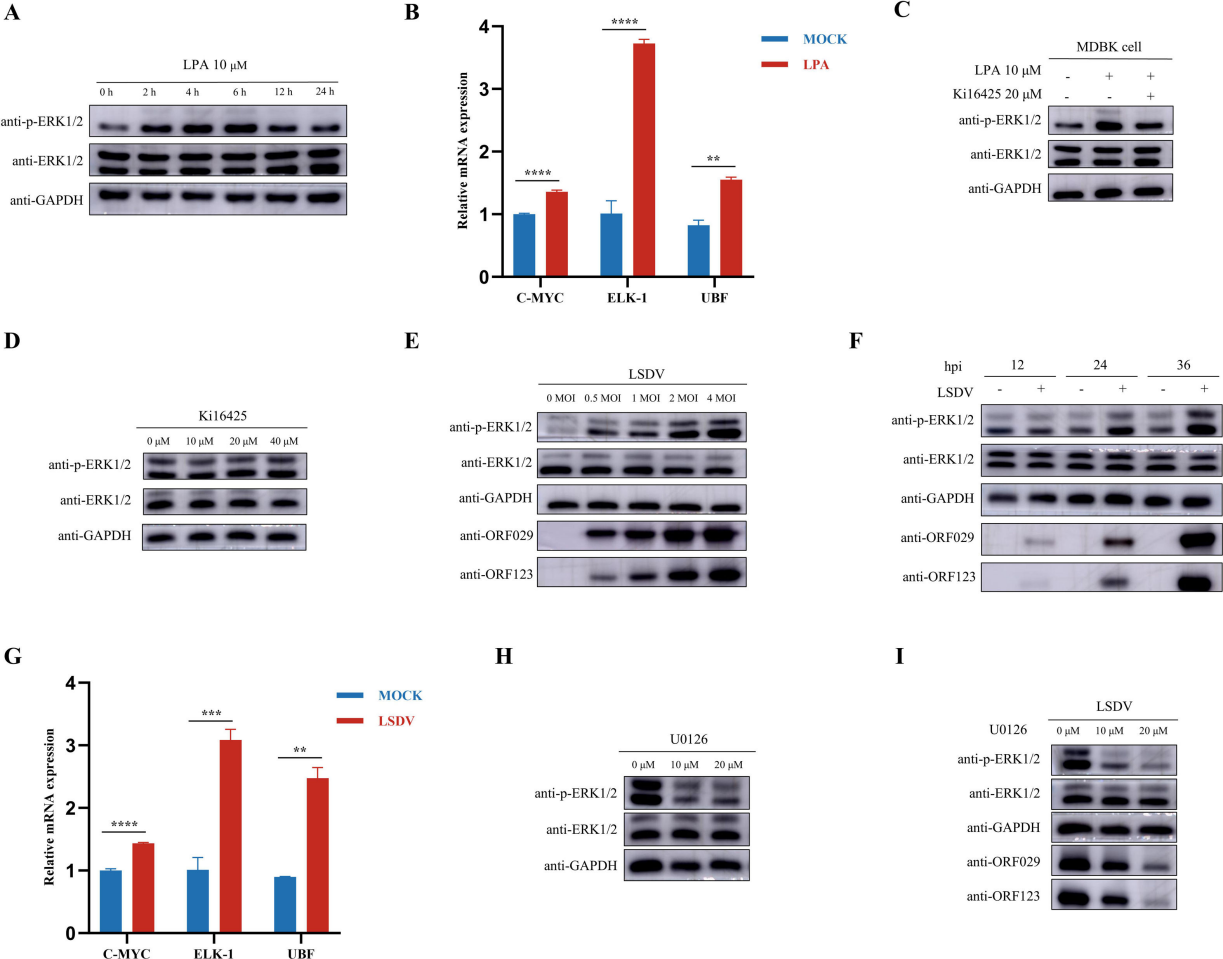
LPA promotes LSDV replication by activating the ERK1/2 signaling pathway. (**A**) MDBK cells were starved for 2 h and then treated with 10 µM LPA. Cell samples were collected at the indicated time, and the expression of total or phosphorylated ERK1/2 was detected by Western blotting. (**B**) MDBK cells were starved for 2 h and then treated with 10 µM LPA for 12 h. Cell samples were collected for qRT-PCR to measure the mRNA expression of C-MYC, ELK-1, and UBF. (**C**) MDBK cells were starved for 2 h and then treated with 10 µM LPA alone or together with 20 µM Ki16425 for 12 h. Cell samples were collected, and the expression of total or phosphorylated ERK1/2 was detected by Western blotting. (**D**) MDBK cells were treated with different doses of Ki16425 (0, 10, 20, and 40 µM) for 12 h. Cell samples were collected, and the expression of total or phosphorylated ERK1/2 was detected by Western blotting. (**E**) MDBK cells were infected with LSDV of different MOI for 24 h. Cell samples were collected, and the expression of total or phosphorylated ERK1/2 was detected by Western blotting. (**F**) MDBK cells were infected with LSDV (0.5 MOI), and cell samples were collected at the indicated time. The expression of total or phosphorylated ERK1/2 and LSDV ORF123 and ORF029 was detected by Western blotting. (**G**) MDBK cells were infected with LSDV (0.5 MOI) for 24 h, and cell samples were collected for qRT-PCR to detect the mRNA expression of C-MYC, ELK-1, and UBF. (**H**) MDBK cells were treated with U0126 for 12 h, and cell samples were collected to detect the expression of total or phosphorylated ERK1/2 by Western blotting. (**I**) MDBK cells were pretreated with different doses of U0126 for 2 h and then infected with LSDV (0.5 MOI) for 24 h. Cell samples were collected to detect the expression of total or phosphorylated ERK1/2, and LSDV ORF123 and ORF029 were detected by Western blotting. *P < 0.05, **P < 0.01, ***P < 0.001, and ****P < 0.0001.

Previous studies have demonstrated that LPA activates the MEK/ERK signaling pathway (20–22). To investigate the influence of LPA on the MEK/ERK signaling pathway in MDBK cells, 10 µM LPA was added to the cell culture supernatant, and the cell samples were collected at the indicated times. As shown in Fig. 8A, LPA significantly promoted ERK1/2 phosphorylation, which gradually decreased. In addition, the expression of the downstream molecules of the MEK/ERK signaling pathway, such as c-Myc, ELK-1, and UBF, was upregulated after LPA treatment (Fig. 8B). A previous study demonstrated that Ki16425, an LPA1/3 receptor inhibitor, reduced LPA-induced activation of the MEK/ERK signaling pathway while acting as a weak stimulator of the MEK/ERK signaling pathway (22). In this study, we found that Ki16425 effectively inhibited the LPA-induced activation of the MEK/ERK signaling pathway in MDBK cells (Fig. 8C). However, the addition of Ki16425 alone did not significantly alter the activation of the MEK/ERK signaling pathway (Fig. 8D). Many studies have demonstrated that the activation of the MEK/ERK signaling pathway is beneficial for viral replication (23, 24); however, the relationship between LSDV replication and the MEK/ERK signaling pathway has not been described. In this study, we found that LSDV infection significantly promotes the phosphorylation of ERK1/2 in a dose- and time-dependent manner (Fig. 8E and F). In addition, the expression of downstream molecules of the MEK/ERK signaling pathway, such as c-Myc, ELK-1, and UBF, was upregulated after LSDV infection (Fig. 8G). To explore the effect of MEK/ERK signaling pathway activation on LSDV replication, a chemically synthesized organic compound, U0126, which specifically inhibits the activation of ERK1/2, was used. MDBK cells were treated with different doses of U0126 and infected with LSDV. Treatment with U0126 significantly suppressed ERK1/2 phosphorylation (Fig. 8H), resulting in a dramatic, dose-dependent decrease in LSDV replication (Fig. 8I). Taken together, these results demonstrate that LPA promotes LSDV replication by activating the MEK/ERK signaling pathway.

### LPA promotes LSDV replication by repressing the host’s innate immune response

**Fig 9:**
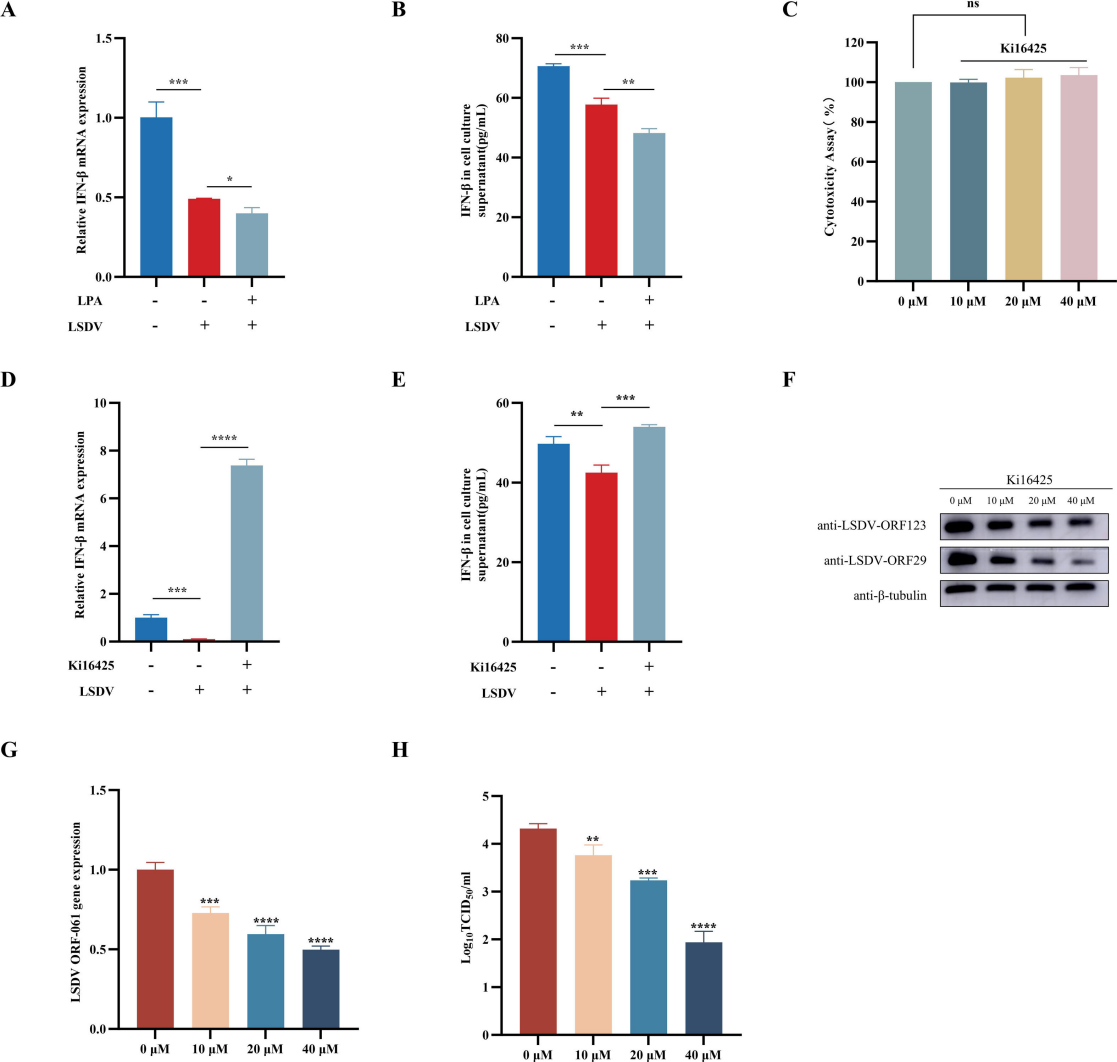
LPA promotes LSDV infection by repressing the host’s innate immune response. (A and B) MDBK cells were starved for 2 h and then treated with 10 µM LPA for 1 h before infection with LSDV (0.5 MOI). The cell samples and culture supernatant were selected for qRT-PCR (A) and ELISA (B) to detect the expression of IFN-β, respectively. (C) CCK-8 assay was used to detect the toxicity of Ki16425 on MDBK cells. (D and E) MDBK cells were infected with LSDV (0.5 MOI) and then treated with 10 µM of Ki16425 for 24 h. The cell samples and culture supernatant were selected for qRT-PCR (D) and ELISA (E) to detect the expression of IFN-β, respectively. (F–H) MDBK cells were infected with LSDV (0.5 MOI) and then treated with different concentrations of Ki16425 (0, 10, 20, and 40 µM) for 24 h. The replication of LSDV was detected by qRT-PCR (B), Western blot (C), and TCID_50_ (D). *P < 0.05, **P < 0.01, ***P < 0.001, and ****P < 0.0001.

A recent study demonstrated that LPA can inhibit type I interferon (IFN) production through LPA1 signaling (25). Next, we investigated the effect of LPA on type I interferon during LSDV infection. As shown in Fig. 9A and B, LSDV infection can significantly reduce the expression and secretion of IFN-β. Meanwhile, LPA treatment can enhance the ability of LSDV to inhibit the expression and secretion of IFN-β (Fig. 9A and B). Next, we examined the influence of Ki16425, an LPA1 signaling inhibitor, on the production of type I IFNs during LSDV infection. First, a CCK-8 assay was performed to evaluate the effect of Ki16425 on cell viability, which showed no significant effect on the viability of MDBK cells at the indicated concentrations (Fig. 9C). As shown in Fig. 9D and E, Ki16425 can significantly promote the expression and secretion of IFN-β during LSDV infection. In this study, we examined the role of Ki16425 in LSDV replication. MDBK cells were pretreated with different doses of exogenous Ki16425 (10, 20, or 40 µM) for 1 h and infected with LSDV (0.5 MOI). At 24 h post-infection, cell samples were collected for WB, qRT-PCR, and TCID_50_ analysis to determine viral replication. The results showed that Ki16425 treatment significantly inhibited LSDV replication in a dose-dependent manner (Fig. 9F through H). Taken together, these results demonstrate that LPA promotes LSDV replication by repressing the host’s innate immune response.

## DISCUSSION

Since its first report in 2019, LSD has spread rapidly throughout China (4). Current methods for controlling LSD rely mainly on isolating and culling infected cattle from affected areas. However, because the spread of LSDV is often incompletely blocked, effective and safe vaccines and antiviral drugs targeting LSDV are urgently required. In this study, we found that TMP269 significantly inhibited LSDV replication in a dose-dependent manner. RNA-seq and untargeted metabolomics analyses demonstrated that TMP269 can reduce LPA metabolism, which was induced by LSDV infection, and is beneficial to LSDV replication by activating the MEK/ERK signaling pathway and suppressing the host innate immune response. Our study illustrates the antiviral mechanism of TMP269 and a novel mechanism by which LSDV inhibits the host’s innate immune response, which provides insights into the development of new preventive or therapeutic strategies targeting altered metabolic pathways.

HDACs are involved in viral replication by regulating host protein acetylation (26, 27). TMP269, a class IIa HDACi (HDAC4/5/7/9), has made significant progress in the study of cancer, neurological disorders, and kidney injury (12, 13). However, few studies have investigated the antiviral effects of TMP269. In this study, we found that TMP269 inhibited LSDV infection in a dose-dependent manner. Further experiments revealed that TMP269 primarily affected the early stages of LSDV replication. RNA-seq analysis showed that several IFN-stimulated genes with antiviral effects, such as IFIT2, IFIT3, and OAS1Y, were significantly upregulated in TMP269-treated group, demonstrating that TMP269 may promote the host innate immune response to repress LSDV replication. In addition, KEGG analysis showed that most of the DEGs were enriched in metabolism-related pathways, demonstrating that TMP269 may affect the host metabolic process to modulate LSDV proliferation.

Despite the lack of autonomous metabolic capacity, viruses can alter host cell metabolism through various mechanisms and pathways to meet their replication energy and material demands (28). For example, the African swine fever virus modulates host energy and amino acid metabolism to promote viral replication (29). *De novo* fatty acid biosynthesis contributes significantly to the establishment of a bioenergetically favorable environment for vaccinia virus infection (30). In this study, we observed that most of the differentially expressed metabolites in the DMSO and TMP269 groups were enriched in lipid metabolism-related pathways, such as glycerophospholipid, linoleic acid, choline, and arachidonic acid metabolism pathways. In addition, most of the differentially expressed metabolites involved in glycerophospholipid metabolism in the TMP269 group were significantly downregulated compared with those in the DMSO group. Studies have revealed that lipid metabolism contributes significantly to the establishment of a favorable environment for viral infection, and genetic or pharmacological inhibition of host lipid metabolism can attenuate viral infection (31–33). Therefore, lipid metabolites may play a crucial role in the life cycle of LSDV, and TMP269 may inhibit LSDV infection by regulating the expression of metabolites involved in host lipid metabolism-related pathways.

Among the differentially expressed metabolites related to glycerophospholipid metabolism, LPA and LPC were significantly altered in the DMSO and TMP269 groups. Previous studies have indicated that the hepatitis C virus (HCV) induces the expression of autotaxin, which hydrolyzes LPC into LPA, resulting in the activation of signaling pathways involving LPA and its receptors that promote HCV replication in hepatic cells (34). LPA is a simple glycerophospholipid composed of a phosphate group, glycerol moiety, and hydrocarbon chain. LPA interacts with six specific G protein-coupled receptors and participates in multiple biological processes, including inflammation, cancer, wound healing, brain development, and neuropathic pain (18, 35, 36). In this study, we observed that LSDV infection promoted LPA expression. In addition, the exogenous addition of LPA significantly promoted LSDV replication. Previous studies have demonstrated that LPA activates the MEK/ERK signaling pathway (20–22). Many studies have demonstrated that the activation of the MEK/ERK signaling pathway is beneficial for viral replication (23, 24). In this study, we found that LPA treatment promoted activation of the MEK/ERK signaling pathway in MDBK cells, which was beneficial for LSDV replication. Treatment of cells with U0126, a specific inhibitor of the MEK/ERK signaling pathway, significantly blocked the MEK/ERK signaling pathway and suppressed LSDV replication. Taken together, these results demonstrate that LPA promotes LSDV replication by activating the MEK/ERK signaling pathway. This confirmed that LSDV manipulated the MEK/ERK signaling pathway and that the MEK/ERK signaling pathway might be a potential target for suppressing LSDV infection.

Recent studies have shown that LPA and its receptors are involved in regulating viral replication by modulating the host’s innate immune response (25, 37, 38). HCV or severe acute respiratory syndrome coronavirus-2 infection can significantly induce the expression of LPA, which can, through the LPA-LPA1 signaling axis, activate ROCK1/2, leading to the phosphorylation of IRF3 Ser97, which in turn inhibits the activation of IRF3 to inhibit the innate immune response (25). In this study, we found that treatment with LPA can enhance the ability of LSDV to inhibit the expression and secretion of IFN-β, while Ki16425 can significantly promote the expression and secretion of IFN-β during LSDV infection. Ki16425 significantly inhibits LSDV replication. These results demonstrated that LPA can significantly promote LSDV replication by inhibiting the host’s innate immune response. Several studies have demonstrated that the MEK/ERK signaling pathway plays a crucial role in immune signaling (39–42). For example, the ERK1/2-TCF-FOS pathway mediates TPL-2 suppression of type I IFN signaling, which is essential for host resistance against intracellular bacterial infections (41). In this study, we found that LPA treatment not only promoted the activation of the MEK/ERK signaling pathway but also inhibited host innate immunity during LSDV infection. However, the regulatory mechanism underlying the activation of the MEK/ERK signaling pathway and the suppression of the innate immune response induced by LPA during LSDV infection still needs to be further explored. Taken together, these results suggest that LPA is a potential antiviral target for the treatment of LSDV infections.

**Fig 10:**
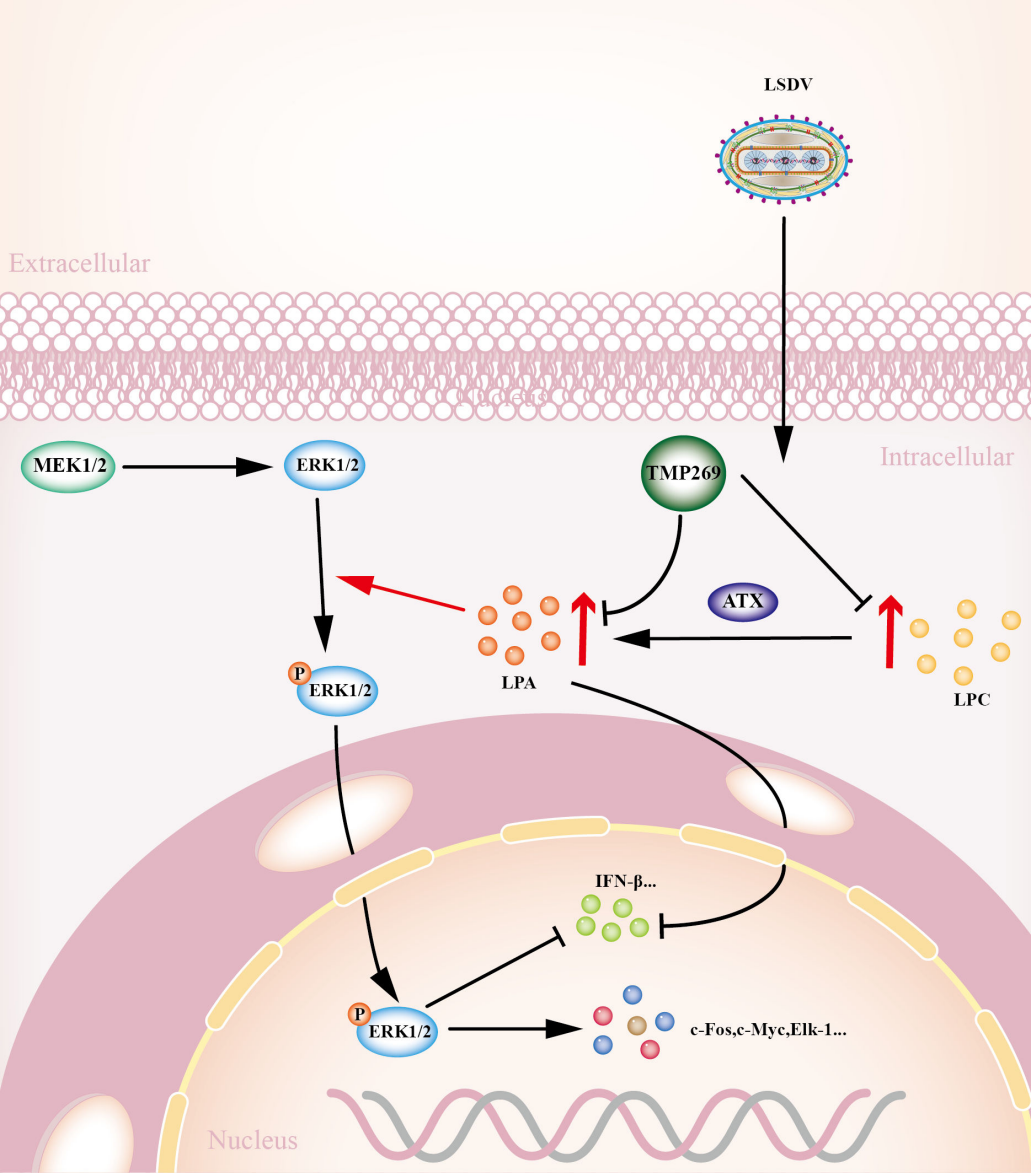
Schematic model of TMP269 suppressing LSDV infection by reducing the expression of LPA induced by LSDV infection.

Based on our findings, we proposed a schematic model of TMP269 inhibition LSDV replication (Fig. 10). In summary, LSDV infection can promote LPA expression to activate the MEK/ERK signaling pathway and suppress the innate immune response, whereas TMP269 treatment can inhibit LPA production, further limiting the promotion of LSDV replication. This study revealed the antiviral mechanism of TMP269 and a novel mechanism by which it manipulates host signaling pathways to promote its own replication, providing important insights for the development of new preventive or therapeutic strategies targeting altered metabolic pathways.

## Data Availability

The RNA-seq data sets presented in this study were deposited in the NCBI repository under accession number PRJNA1027065.

## SUPPLEMENTAL MATERIAL

Fig. S1 jvi.01827-24-s0001.tif TMP269 significantly inhibits GTPV infection.

Fig. S2 jvi.01827-24-s0002.tif KEGG classification map of differential metabolites in the DMSO group compared with the MOCK group.

Fig. S3 jvi.01827-24-s0003.tif KEGG classification map of differential metabolites in the TMP269 group compared with the DMSO group.

Fig. S4 jvi.01827-24-s0004.tif Chemical structure of TMP269.

Supplemental legends jvi.01827-24-s0005.docx Legends for supplemental tables and figures.

Table S1 jvi.01827-24-s0006.xlsx List of differentially expressed genes in the TMP269 group compared with the DMSO group.

Table S2 jvi.01827-24-s0007.xlsx List of differential metabolites in the DMSO group compared with the MOCK group.

Table S3 jvi.01827-24-s0008.xlsx List of differential metabolites in the TMP269 group compared with the DMSO group.

ASM does not own the copyrights to Supplemental Material that may be linked to, or accessed through, an article. The authors have granted ASM a non-exclusive, world-wide license to publish the Supplemental Material files. Please contact the corresponding author directly for reuse.
