# Supplementary figures and images for: Class IIa histone deacetylase (HDAC) inhibitor TMP269 suppresses lumpy skin disease virus replication by regulating host lysophosphatidic acid metabolism
Source: J Virol. 2025 Jan 22;99(2):e01827-24. doi: 10.1128/jvi.01827-24 (PMC11852836; doi:10.1128/jvi.01827-24)

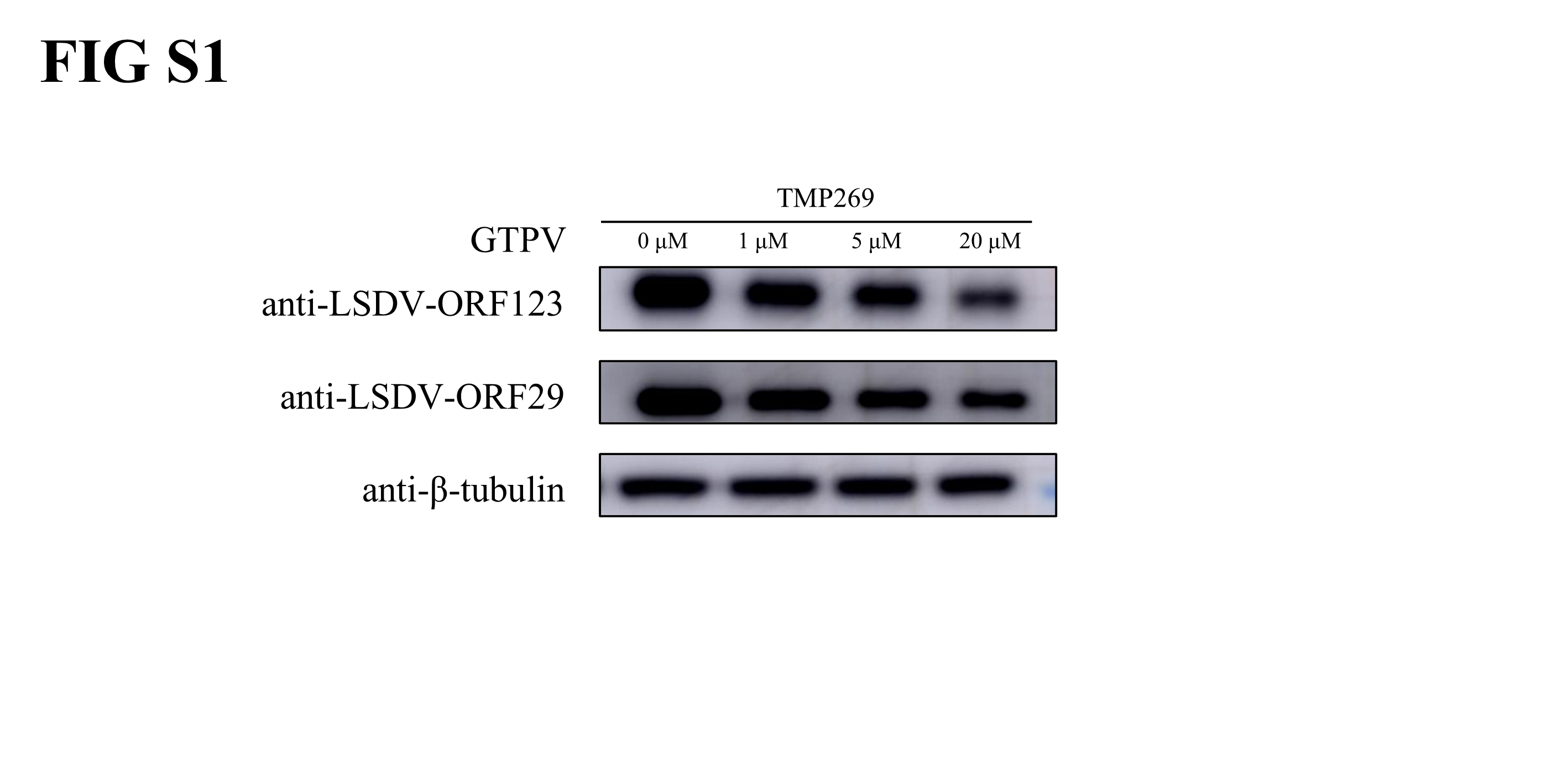

Supplement: Fig. S1 — TMP269 significantly inhibits GTPV infection. [file jvi.01827-24-s0001.tif]

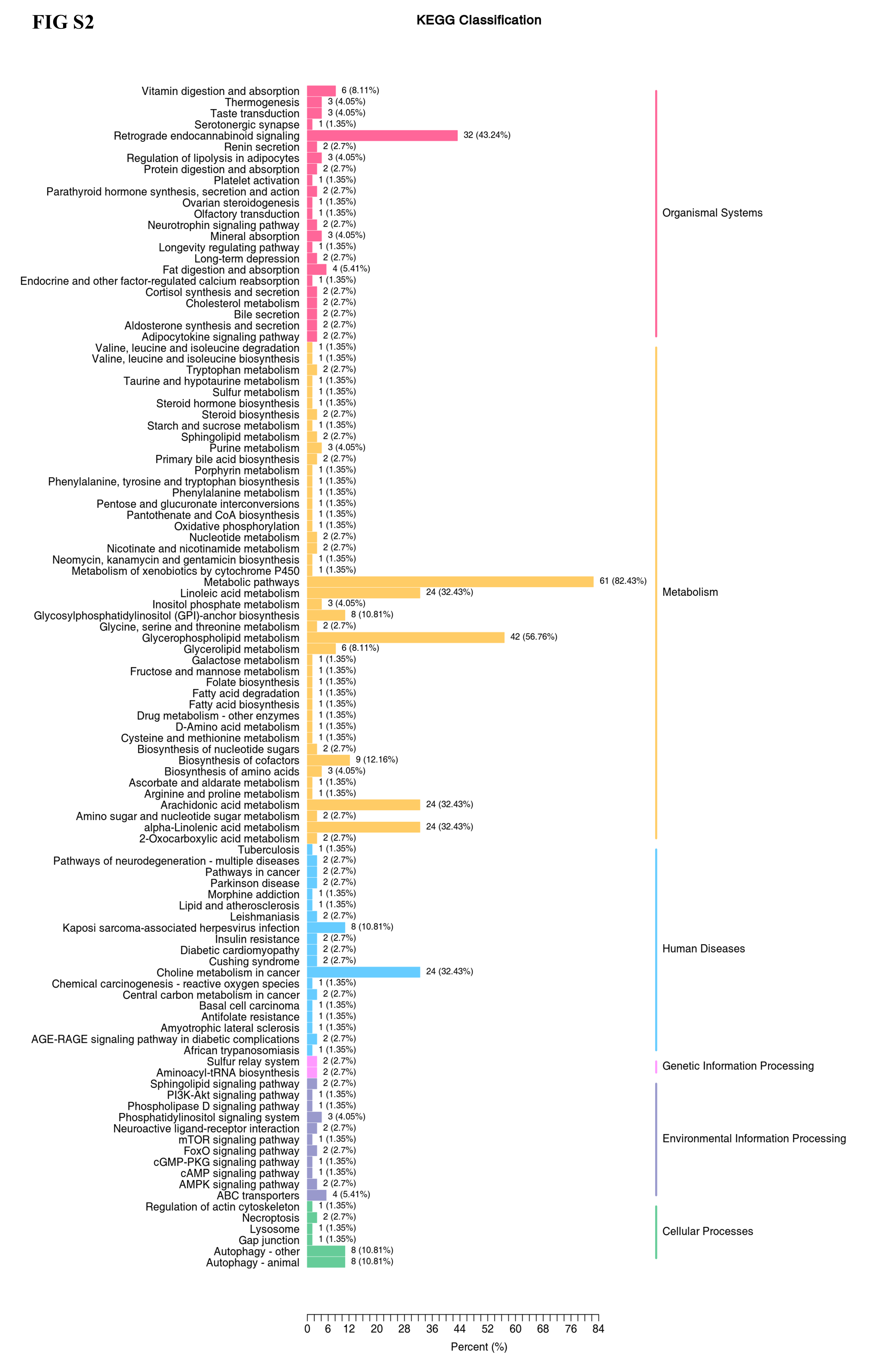

Supplement: Fig. S2 — KEGG classification map of differential metabolites in the DMSO group compared with the MOCK group. [file jvi.01827-24-s0002.tif]

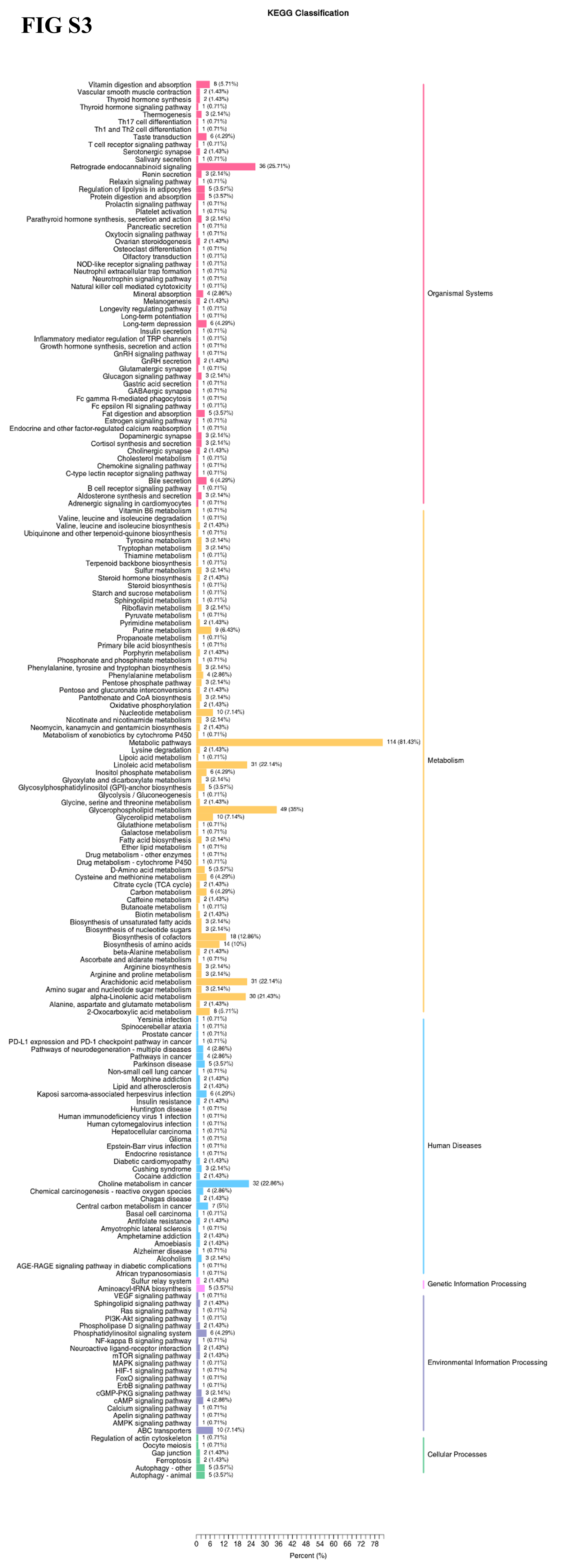

Supplement: Fig. S3 — KEGG classification map of differential metabolites in the TMP269 group compared with the DMSO group. [file jvi.01827-24-s0003.tif]

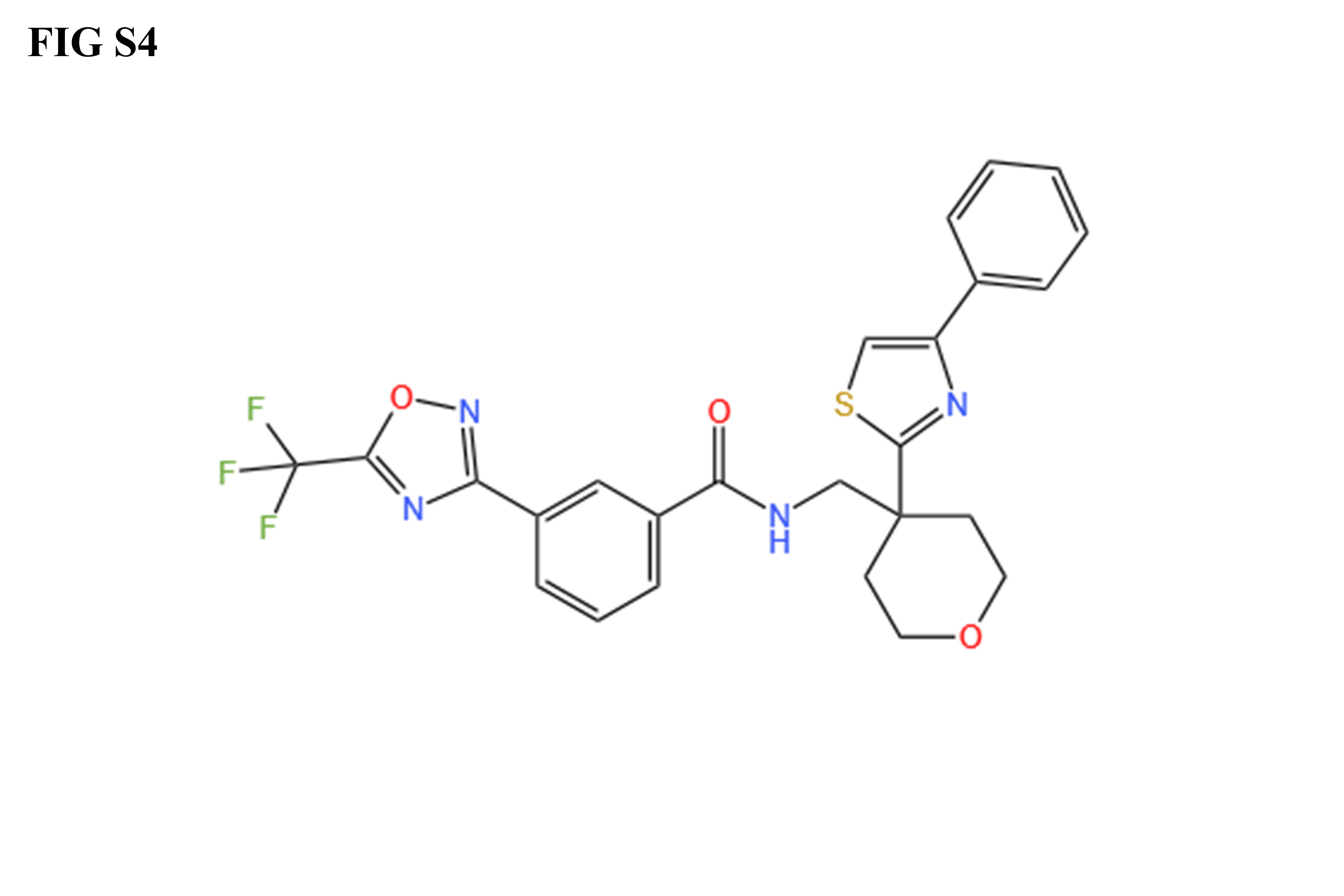

Supplement: Fig. S4 — Chemical structure of TMP269. [file jvi.01827-24-s0004.tif]
