# Supplementary material for: Class IIa histone deacetylase (HDAC) inhibitor TMP269 suppresses lumpy skin disease virus replication by regulating host lysophosphatidic acid metabolism
Source: J Virol. 2025 Jan 22;99(2):e01827-24. doi: 10.1128/jvi.01827-24 (PMC11852836; doi:10.1128/jvi.01827-24)
Supplement: Supplemental legends — Legends for supplemental tables and figures. [file jvi.01827-24-s0005.docx]

**SUPPLEMENTAL FILE**

**TABLE S1** List of differentially expressed genes in the TMP269 group compared with the DMSO group.

**TABLE S2** List of differential metabolites in the DMSO group compared with the MOCK group.

**TABLE S3** List of differential metabolites in the TMP269 group compared with the DMSO group.

**FIG S1** TMP269 significantly inhibits GTPV infection. Effect of TMP269 on GTPV replication. The cells were infected with GTPV (AV41 strain) and simultaneously treated with different concentrations of TMP269 (10, 20, and 30 μM) for 24 h. The replication of GTPV was detected by Western blot.

**FIG S2** KEGG classification map of differential metabolites in the DMSO group compared with the MOCK group. The ordinate is the name of KEGG metabolic pathway, and the abscissa is the number of differential metabolites annotated to the pathway and the proportion of the number to the total number of metabolites annotated.

**FIG S3** KEGG classification map of differential metabolites in the TMP269 group compared with the DMSO group. The ordinate is the name of KEGG metabolic pathway, and the abscissa is the number of differential metabolites annotated to the pathway and the proportion of the number to the total number of metabolites annotated.

**FIG S4** Chemical structure of TMP269.
